# Sublingual Dripping Pill Formulation of *Ganoderma amboinense* Fruiting Body Extract Attenuates CCl_4_-Induced Liver Fibrosis via Multi-Pathway Regulation

**DOI:** 10.3390/cimb47090697

**Published:** 2025-08-28

**Authors:** Chin-Feng Liu, Chong-Ming Pan, Chun-Lin Lee

**Affiliations:** 1 Continuing Education Program of Food Biotechnology Applications, National Taitung University, Taitung 95092, Taiwan; cfliu@nttu.edu.tw; 2 Department of Life Science, National Taitung University, 369, Sec. 2, University Rd., Taitung 95092, Taiwan; gh851005@gmail.com

**Keywords:** *Ganoderma amboinense*, ganoderic acid A, sublingual dripping pill, liver fibrosis, bioavailability

## Abstract

Liver fibrosis remains difficult to treat, in part because many hepatoprotective triterpenoids suffer from poor oral bioavailability and lack of optimized delivery formats. *Ganoderma amboinense* is a rare “antler” reishi species long valued in Eastern traditions yet scarcely studied for its phytochemical and pharmacological potential. Here, we report the first investigation of an ethanol-extracted *G. amboinense* sublingual dripping pill formulation (GDP) in a carbon-tetrachloride (CCl_4_)–induced mouse model of liver fibrosis. Mice treated with GDP at one- and five-times the human equivalent dose were compared to groups receiving unprocessed *G. amboinense* powder (GP) or purified ganoderic acid A (GA-A). GDP significantly prevented CCl_4_-induced weight loss and hepatomegaly, normalizing liver-to-body weight ratios and serum AST/ALT activities (*p* < 0.05). Histological evaluation showed that GDP markedly reduced hepatocellular necrosis and collagen deposition, restoring tissue architecture. Furthermore, GDP suppressed hepatic expression of pro-inflammatory cytokines (TNF-α, IL-6, IL-1β, COX-2) and profibrotic markers (TGF-β1, CTGF, α-SMA) to levels comparable with or superior to GA-A. These results demonstrate that a dripping pill dosage form can effectively deliver *G. amboinense* triterpenoids and unlock their hepatoprotective activity, supporting further development of GDP as a novel liver-support nutraceutical.

## 1. Introduction

Liver disease arises from diverse etiologies—including drug-induced injury [1], viral hepatitis [2], alcoholic liver disease [3], and non-alcoholic fatty liver disease [4]—and, if left untreated, chronic inflammation progresses to hepatic fibrosis and ultimately cirrhosis. Hepatic fibrosis is characterized by repeated injury-repair cycles leading to activation of hepatic stellate cells (HSCs) and excessive accumulation of extracellular matrix (ECM) proteins such as type I/III collagen and fibronectin (Fn) [5]. Under physiological conditions, ECM synthesis and degradation remain in equilibrium; when ECM deposition outpaces degradation, irreversible fibrosis ensues. Current anti-fibrotic strategies target inhibition of ECM accumulation and induction of HSC apoptosis [6].

Fungal-derived medicines have played an important role in both traditional and modern healthcare, offering a wide range of bioactive compounds with anti-inflammatory, antioxidant, immunomodulatory, and hepatoprotective effects. In particular, basidiomycete fungi such as *Ganoderma*, *Cordyceps*, and *Antrodia* have been extensively studied for their therapeutic potential in chronic liver diseases and other metabolic disorders. Among these, *Ganoderma* species are especially valued for their triterpenoids and polysaccharides, which have been shown to modulate key fibrogenic pathways and protect against liver injury [7]. The carbon tetrachloride (CCl_4_)–induced mouse model is one of the most widely used and well-characterized models for evaluating hepatoprotective agents and was appropriate for the present fibrosis-induction design. In hepatocytes, CCl_4_ is bioactivated by cytochrome P450 (CYP) enzymes into trichloromethyl (•CCl_3_) and trichloromethyl peroxy (•CCl_3_OO) radicals, which initiate lipid peroxidation and oxidative stress, trigger pro-inflammatory cytokine release, and drive HSC activation and ECM overproduction [8].

*Ganoderma* species have long been employed in traditional Asian medicine to promote liver health, enhance immunity, and delay aging [9]. Research on the genus *Ganoderma* has overwhelmingly focused on *Ganoderma lucidum*, whose triterpenoid constituents—particularly ganoderic acids—have been extensively characterized for hepatoprotective and anti-inflammatory activities [10]. By contrast, *Ganoderma amboinense* remains relatively understudied despite its unique biology and chemical profile. Of over 2000 known *Ganoderma* species, *G. amboinense* is notable for its antler-shaped fruiting bodies that develop under elevated CO_2_ conditions, where they accumulate exceptionally high levels of lanostane-type triterpenoids—most prominently ganoderic acid A—and β-1,3-glucan polysaccharides at the apex [11,12,13]. These bioactive compounds scavenge reactive oxygen species (ROS), suppress inflammation, and inhibit fibrogenic signaling pathways [14,15]. Although *G. lucidum* remains the most widely studied *Ganoderma* species, *G. amboinense* has drawn increasing attention due to its distinct phytochemical profile and rare morphology. In particular, it produces unique lanostane-type triterpenoids such as ganoderiol F and ganoderic acid X, which exhibit hepatoprotective and anti-proliferative activities not commonly found in *G. lucidum* [11,16,17]. Previous studies have shown that *G. amboinense* extracts can protect against acetaminophen-induced liver injury in mice [18], suggesting its therapeutic potential in hepatic diseases. Furthermore, its antler-shaped fruiting body is highly valued in East Asian traditional medicine, yet remains scientifically underexplored. Therefore, this study aims to not only optimize the bioactive extraction and delivery format but also to scientifically validate the pharmacological properties of this culturally important and underutilized species. *Ganoderma amboinense*, a lesser-known species within the *Ganoderma* genus, has garnered interest in recent years due to its unique morphology and cultural appeal, particularly among East Asian populations. Unlike the extensively studied *Ganoderma lucidum*, *G. amboinense* remains underexplored, and its phytochemical composition, extraction protocols, and functional dosage forms are still largely undefined. The distinct appearance of *G. amboinense* has long been admired in traditional medicine contexts; however, scientific investigations into its bioactivity and medicinal potential have lagged behind. Moreover, emerging evidence suggests that the bioavailability and pharmacological efficacy of *Ganoderma* species can be significantly influenced by formulation type and processing techniques. In light of this, the present study focuses on the extraction and functional characterization of *G. amboinense*, aiming to explore its bioactive lanostane-type triterpenoids and to assess their potential biological effects. This investigation represents a novel contribution to the field, offering valuable insights into a species that has been historically appreciated but scientifically neglected. When comparing *Ganoderma amboinense* and *Ganoderma lucidum*, several key differences in phytochemistry and pharmacology emerge. *G. lucidum* is well-known for its triterpenoid-rich profile—particularly ganoderic acids—which have been extensively studied for their hepatoprotective, anti-inflammatory, and antifibrotic properties [19,20]. In contrast, *G. amboinense* has recently been shown to contain novel lanostane-type triterpenoids with distinct structural features [11], yet their functional bioactivity remains poorly understood. Given the highly lipophilic nature and poor oral bioavailability of ganoderic acids, particularly GA-A, there is strong scientific rationale for developing delivery systems that enhance their systemic exposure. Sublingual administration can bypass first-pass metabolism and gastrointestinal degradation, leading to improved absorption and bioavailability [10,16,17]. Moreover, formulation optimization allows for the protection of oxidation-prone triterpenoids and better control over release kinetics, which are critical for maintaining pharmacological activity during storage and administration [10,21,22]. These considerations support the exploration of innovative dosage forms for *G. amboinense* triterpenoids to maximize their therapeutic potential.

Sublingual dripping pill formulations harness the dense sublingual vasculature to bypass gastrointestinal degradation and achieve rapid, precise delivery of lipophilic actives [23,24]. By incorporating PEG 4000 at optimized ratios, release kinetics can be tuned from immediate to sustained profiles, while co-formulating red quinoa (RQ) bran extract (*Chenopodium formosanum*) provides potent antioxidant protection [25], preventing oxidative degradation of sensitive lanostane triterpenoids during processing and storage [26]. Together, these excipients enable a stable, controllable dripping pill system for reliable sublingual delivery of *Ganoderma amboinense* triterpenoids.

In this study, we optimized a one-pot extraction of GA to maximize yields of GA-A and β-1,3-glucan and scaled the process to a 300 L pilot plant. This one-pot extraction approach was specifically optimized to enable the simultaneous recovery of ganoderic acid A (GA-A) and β-1,3-glucan from *G. amboinense*. Such co-extraction streamlines processing, reduces solvent use, and minimizes degradation of thermolabile or oxidation-sensitive compounds. Importantly, ganoderic acid A (IUPAC name: (3β,7β,15α)-3,7,15-trihydroxy-11,23-epoxy-lanost-8,16-dien-26-oic acid) is a lanostane-type triterpenoid with well-documented hepatoprotective and anti-fibrotic activities, while β-1,3-glucan is a bioactive polysaccharide known for its immunomodulatory, antioxidant, and tissue-repair–supporting properties. The co-presence of these two complementary bioactives may provide additive or synergistic benefits, thereby enhancing the therapeutic potential of the final formulation. We then prepared a granulated extract powder and an innovative sublingual dripping pill (GDP) using PEG 4000/RQ phenolics as composite excipients. Finally, we conducted a head-to-head comparison of GDP and purified GA-A in a CCl_4_-induced mouse fibrosis model. Endpoints included body and liver weight ratios, serum AST/ALT, histopathology (H&E, Sirius Red), inflammatory cytokines (IL-1β, IL-6, COX-2, TNF-α), and fibrotic markers (TGF-β1, CTGF, α-SMA). We hypothesize that the novel GDP formulation will demonstrate superior GA-A bioavailability and anti-fibrotic efficacy compared to conventional powder and pure compound forms, thereby informing preclinical pharmacokinetic and safety evaluations. While the current study provides functional evidence supporting improved performance of the GDP formulation, direct pharmacokinetic or safety studies were not conducted, and confirmation of bioavailability advantages will require future dedicated investigations.

## 2. Materials and Methods

### 2.1. Chemicals

All solvents and standards were of analytical or HPLC grade. Ethanol (95%) for co-extraction was purchased from ECHO CHEMICAL Co., Ltd. (Miaoli, Taiwan); methanol and acetonitrile for HPLC mobile phases were obtained from Avantor Inc. (Radnor, PA, USA); and formic acid (≥98%) was supplied by Honeywell Taiwan Ltd. (New Taipei, Taiwan). Reference standards of ganoderic acid A (>98% purity) were sourced from Chengdu Must Bio-Technology Co. Ltd. (Chengdu, Sichuan, China), while rutin and β-1,3-glucan standards were acquired from Sigma-Aldrich Co. (St. Louis, MO, USA). Carbon tetrachloride (≥99%) for induction of liver fibrosis was obtained from ALPS GHEM Co., Ltd. (New Taipei, Taiwan) and diluted in olive oil (1:4, *v*/*v*) immediately before use. Total protein concentrations were determined using the BCA Protein Assay Kit (Cat. No. 23225; Merck KGaA, Millipore, Darmstadt, Germany). Serum levels of TNF-α and IL-6 were measured with ELISA kits (Cat. Nos. 50349-MNAE and 50136-MNAE; Sino Biological Inc., North Wales, PA, USA), and CTGF concentrations with the CTGF ELISA Kit (LS-G26584; Lifespan Biosciences Inc., Seattle, WA, USA). For immunoblotting and immunohistochemistry, we used rabbit anti-mouse TNF-α polyclonal antibody (AB2148P; EMD Millipore, Temecula, CA, USA), mouse anti-rat IL-6 monoclonal antibody (sc-57315; Santa Cruz Biotechnology, Dallas, TX, USA), anti-TGF-β1 polyclonal antibody (39303; Signalway Antibody, College Park, MD, USA), anti-α-smooth muscle actin (ACTA2) monoclonal antibody (04-1094; EMD Millipore, Temecula, CA, USA), and anti-CTGF antibody (FNab02054; Wuhan Fine Biotech, Wuhan, Hubei, China), each used at the manufacturer’s recommended dilution.

### 2.2. Preparation of Test Substances

#### 2.2.1. Extraction of *G. amboinense* Fruiting Body

The *Ganoderma amboinense* fruiting bodies (antler-type reishi) used in this study were provided by GE Health R&D Co., Ltd. (Taitung, Taiwan). The strain is a proprietary culture maintained in the company collection and used to produce the characteristic antler-shaped fruiting bodies. This extraction protocol was developed based on preliminary pilot-scale extractions (1–3 kg scale) performed in-house, which demonstrated consistent yields of ganoderic acid A and good product stability, supporting the decision to proceed with 300-L scale-up.

*Ganoderma amboinense* fruiting bodies contain both water-soluble constituents (e.g., polysaccharides) and lipid-soluble constituents (e.g., triterpenoids). To ensure comprehensive recovery of bioactive compounds, water and ethanol extractions were performed separately and then combined. A 300-L extractor (YC-MEC-300, YENCHEN MACHINERY CO., LTD., Taoyuan, Taiwan) was charged with 23 kg of *G. amboinense*, followed by an initial 2-h aqueous extraction with 200 L of water at 80 °C and a second 1-h aqueous extraction with 100 L of water at 80 °C. The two aqueous extracts were combined and vacuum-concentrated (VC-200, YENCHEN MACHINERY CO., LTD., Taoyuan, Taiwan). This was followed by two ethanolic extractions (120 L and 80 L of 95% ethanol, at 70 °C for 2 h and 1 h, respectively), with filtrates pooled and concentrated to yield the final extract.

#### 2.2.2. Preparation of Dripping Pill Formulations

The concentrated *G. amboinense* extract was lyophilized to yield a dry powder. For the sublingual dripping pill (GDP), the extract powder was blended with PEG 4000 (50% *w*/*w*) and red quinoa phenolic extract (20% *w*/*w*) at 70 °C until homogeneous, then passed through a 20-mesh sieve. The molten mixture was formed into uniform spherical droplets using a drip-casting machine (LUNG SHENG Precision Industries Co., Ltd., Kaohsiung, Taiwan) equipped with a 3.0 mm nozzle, operating at a constant feed rate (5 mL/min) and temperature (70 °C). Droplets solidified within approximately 2 min at room temperature due to PEG 4000 crystallization. Residual surface oil was removed by centrifugation and blotting. Weight uniformity testing of 30 randomly selected units yielded a mean weight of 30 mg, meeting pharmacopeial requirements for solid dosage forms. The inclusion of red quinoa phenolics was specifically intended to inhibit oxidative degradation of ganoderic acid A during storage and thereby prolong product shelf life.

#### 2.2.3. Quantitative Analysis of Active Constituents

GDP samples were dissolved in methanol (1:4, *w*/*v*), diluted, and filtered (0.45 μm). Ganoderic acid A was quantified by HPLC (Model L-2130, Hitachi Co., Tokyo, Japan) on a YMC-Triart C_18_ column (250 × 4.6 mm, 5 μm) with a gradient of acetonitrile and 0.1% formic acid in water (30–40.7% A over 25 min, to 100% A by 40 min; 1 mL/min; 253 nm; 40 °C), using a previously described method with slight modifications [27]. Rutin from the red quinoa phenolic extract was analyzed using a modified HPLC method based on Lin et al. [15] with an Ascentis^®^ C18 column (250 × 4.6 mm, 5 μm). The mobile phase consisted of acetonitrile (solvent A) and 0.1% formic acid in water (solvent B) with the following gradient: 0–20 min, 17% A; 20–25 min, 17–65% A; 25–35 min, 65% A; 35–40 min, 65–100% A; 40–45 min, 100% A; 45–50 min, 100–17% A; 50–55 min, 17% A. The flow rate was 1 mL/min, the detection wavelength was 250 nm, the injection volume was 20 μL, and the column temperature was maintained at 40 °C.

### 2.3. In Vivo Evaluation in a CCl_4_-Induced Mouse Fibrosis Model

#### 2.3.1. Animal Housing and Group Allocation

Male BALB/c mice (8 weeks old, weighing approximately 20–25 g) were obtained from the National Laboratory Animal Center in Taipei, Taiwan. Animals were maintained under controlled conditions (24 ± 1 °C, 60% relative humidity) on a 12 h light–dark cycle (lights on from 08:00 to 20:00), with free access to standard chow and water throughout the study. Mice were randomized into six experimental groups (*n* = 7 per group). The group size was selected to ensure sufficient statistical power to detect meaningful differences in key endpoints while adhering to the 3Rs principle (Replacement, Reduction, and Refinement) for ethical animal use. The normal control group received intraperitoneal injections of olive oil vehicle only. The fibrosis control group was administered carbon tetrachloride (CCl_4_) dissolved in olive oil at a dose of 0.4 mL per kilogram of body weight, without any test article. Treatment groups received CCl_4_ in the same manner and, in addition, one of the following by oral gavage once daily: a *Ganoderma amboinense powder* (GP) formulation at 410 mg/kg/day; *Ganoderma amboinense* dripping pills (GDP) at either 92.25 mg/kg/day or 461.25 mg/kg/day; or purified ganoderic acid A (GAA) at 1.07 mg/kg/day. Dosages for mice were calculated according to the U.S. Food and Drug Administration (FDA) body surface area conversion guidance [28], using the formula: Mouse dose (mg/kg) = [Human daily dose (mg) ÷ 60] × 12.3, where 60 kg represents the reference adult human body weight and 12.3 is the body surface area scaling factor for mice. Animals were observed daily for appearance, activity, and food intake to monitor potential adverse effects.

#### 2.3.2. Fibrosis Induction Protocol

The CCl_4_-induced hepatic fibrosis mouse model was established based on a previously reported method with modifications [29]. The entire experiment lasted 6 weeks. From Day 1, treatment groups received daily oral administration of GDP, GP, or GAA. Starting in Week 3, all groups except the normal control (NOR) received intraperitoneal injections of CCl_4_ (0.4 μL/g body weight in olive oil) three times per week for six consecutive weeks, for a total of 18 injections. This prospective design allowed GDP, GP, or GAA to be administered as a prophylactic intervention, enabling evaluation of their ability to prevent or attenuate fibrosis development. At the end of Week 6, all animals were sacrificed for histopathological, biochemical, and molecular analyses.

#### 2.3.3. Sacrifice and Sample Collection

At the end of the sixth week, mice were fasted overnight (14 h), weighed, and euthanized by CO_2_ asphyxiation. Blood was collected by cardiac puncture, allowed to clot at room temperature for two hours, then centrifuged at 3000× *g* for 10 min (Z326K, Hermle Labortechnik, Wehingen, Germany) to obtain serum, which was stored at –20 °C for later biochemical assays. Livers were excised, blotted dry, and weighed. Representative portions were fixed in 10% neutral-buffered formalin for histological processing, while the remaining tissue was rinsed with 0.9% saline and frozen at –80 °C for biochemical and molecular analyses.

#### 2.3.4. Serum Biochemistry

Serum activities of aspartate aminotransferase (AST) and alanine aminotransferase (ALT) were measured by a certified clinical laboratory using an automated biochemical analyzer (Beckman Coulter AU-700, Beckman Coulter Inc., Brea, CA, USA).

#### 2.3.5. Histopathological Assessment

Formalin-fixed liver specimens were paraffin-embedded and sectioned at 3 μm thickness. Sections were stained with hematoxylin and eosin to evaluate general morphology and with picrosirius red to visualize collagen deposition. Stained sections were examined under light microscopy (EX 20, Sunny Optical Technology Co. Ltd., Yuyao, Zhejiang Province, China) at magnifications of 40×, 100×, and 400×, and representative images were captured for semi-quantitative assessment of fibrosis.

#### 2.3.6. Liver Homogenate Preparation and Protein Quantification

Frozen liver tissue (~0.1 g) was homogenized in 1 mL ice-cold phosphate-buffered saline (0.1 M, pH 7.4) and centrifuged at 10,000× *g* for 10 min at 4 °C. The supernatant was collected, and the procedure was repeated until the homogenate was free of particulate debris. Total protein concentration in the final supernatant was determined by the bicinchoninic acid (BCA) assay, using bovine serum albumin standards and measuring absorbance at 562 nm after a 30 min reaction at 37 °C.

#### 2.3.7. Quantification of Inflammatory and Fibrotic Biomarkers

Levels of tumor necrosis factor-α (TNF-α), interleukin-6 (IL-6), connective tissue growth factor (CTGF), transforming growth factor-β1 (TGF-β1), and α-smooth muscle actin (α-SMA) in liver homogenates were measured by enzyme-linked immunosorbent assay (ELISA). Homogenate samples (100 μL) were incubated in antibody-coated 96-well plates at 37 °C for 1 h, washed three times with PBS-Tween 20, then incubated with primary antibody (100 μL) for an additional hour. After washing, wells were incubated with horseradish peroxidase-conjugated secondary antibody (100 μL) for 1 h, followed by incubation with tetramethylbenzidine substrate for 15 min. The reaction was terminated with 2 N sulfuric acid, and absorbance was read at 450 nm. Biomarker concentrations were interpolated from standard curves.

### 2.4. Statistical Analysis

All quantitative data are expressed as mean ± standard deviation. Group comparisons were performed by one-way analysis of variance followed by Duncan’s multiple range test using SPSS version 22.0. Differences were considered statistically significant at *p* < 0.05.

## 3. Results

### 3.1. Ganoderic Acid a of Ganoderma amboinense Fruiting Bodies Dripping Pill Formulation

**Figure 1:**
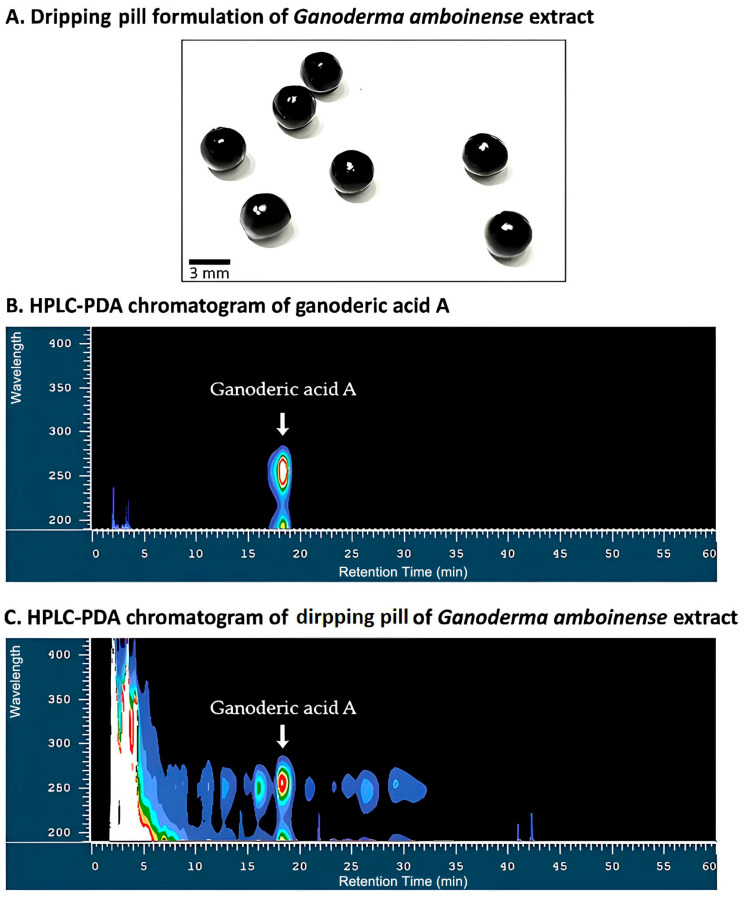
Appearance and HPLC–PDA profile of *Ganoderma amboinense* dripping pills. (**A**) Photograph of the sublingual dripping pill formulation containing *G. amboinense* extract. (**B**) HPLC–PDA chromatogram of the ganoderic acid A reference standard, showing a single, well-resolved peak at approximately 17.5 min and its characteristic UV spectrum. (**C**) HPLC–PDA chromatogram of the *G. amboinense* dripping pill extract, with a peak at the same retention time and identical UV absorbance profile, confirming the presence of ganoderic acid A within the complex matrix. Data were recorded using photodiode array detection over 200–400 nm.

A total of 23 kg of *Ganoderma amboinense* fruiting bodies were subjected to ethanol extraction using 120 L and 80 L of 95% ethanol at 70 °C for 2 h and 1 h, respectively. The resulting extract was vacuum-concentrated and freeze-dried. The extraction of *Ganoderma amboinense* fruiting bodies yielded 12.4% (*w*/*w*) dried extract. One kg of the dried extract was subsequently blended with polyethylene glycol 4000 (1:1, *w*/*w*) and supplemented with 20% (*w*/*w*) red quinoa (*Chenopodium formosanum*) phenolic extract as a stabilizing excipient. The mixture was then heated to 70 °C, passed through a 20-mesh sieve, and molded into sublingual dripping pills. This pilot-scale process yielded 2.3 kg of dripping pill formulation (GDP) (Figure 1A). The chemical identity and purity of ganoderic acid A within the final GDP product were verified by high-performance liquid chromatography with photodiode array detection (HPLC-PDA). As illustrated in Figure 1B,C, both the reference standard ganoderic acid A and the GDP sample exhibited a well-resolved peak at approximately 17.5 min, with consistent UV absorbance spectra spanning 200–400 nm. These findings confirm the retention and integrity of ganoderic acid A within the formulated dripping pills, despite the presence of other co-extracted compounds. Quantitative HPLC analysis further revealed that the final GDP product contained 2.935 mg/g of ganoderic acid A. The application of PDA detection not only ensured accurate chromatographic identification but also provided spectral fingerprinting for compound verification, highlighting the robustness of the formulation process in preserving triterpenoid bioactivity. In addition, HPLC analysis confirmed the presence of rutin derived from the red quinoa phenolic extract, with a quantified content of 220 mg/kg in the final GDP product. The formulated GDP was a uniform, spherical solid dosage form with a diameter of approximately 3.0 mm and a mean weight of 30 mg. The surface was smooth and dark brown in color, with no visible cracks or deformation. These characteristics support reproducibility and quality assessment of the formulation.

### 3.2. Body Weight, Liver Weight, and Liver-to-Body Weight Ratio

Initial body weights were comparable across all groups (Week 0; *p* > 0.05). From Week 2 onward, the CCl_4_ control group exhibited significantly reduced weight gain relative to the NOR (*p* < 0.05) (Table 1). By Week 8, mice treated with GDP at both 1× and 5× doses, as well as with purified ganoderic acid A (GAA), showed significantly greater weight gain than the CCl_4_ group (*p* < 0.05), with the GDP 5× and GAA groups performing equivalently; the unextracted powder group (GP) demonstrated only a non-significant trend toward weight preservation (*p* > 0.05). CCl_4_ administration also induced marked hepatomegaly, as evidenced by significant increases in absolute liver weight and liver-to-body weight ratio compared with NOR (*p* < 0.05; Table 1). All treatment groups (GP, GDP 1×, GDP 5×, GAA) significantly reversed these increases relative to the CCl_4_ control (*p* < 0.05), with no significant differences among treatment groups (*p* > 0.05). These data indicate that the sublingual dripping pill formulation effectively mitigates CCl_4_-induced hepatic enlargement and weight loss. As expected, the NOR group receiving only olive oil showed baseline physiological values, as olive oil does not induce hepatotoxicity. The therapeutic effects observed in the GAA and GDP groups represent recovery from CCl_4_-induced liver injury, rather than exceeding normal levels. No abnormal clinical signs or mortality were observed in any treatment group during the study.

### 3.3. Serum AST and ALT Activities

Serum aspartate aminotransferase (AST) and alanine aminotransferase (ALT) activities were significantly elevated in the CCl_4_ group compared with NOR (*p* < 0.05) (Table 2), indicating hepatocellular injury. Oral administration of GDP (1×, 5×) and GAA each led to significant reductions in AST (–38%, –45%, –43%, respectively) and ALT (–35%, –50%, –48%) activities compared with CCl_4_ (*p* < 0.05), with GDP 5× showing the greatest effect. The GP group exhibited only a non-significant downward trend. These findings demonstrate that the dripping pill dosage markedly improves liver function markers compared to the powder form.

### 3.4. Histopathological and Fibrotic Assessment

**Figure 2:**
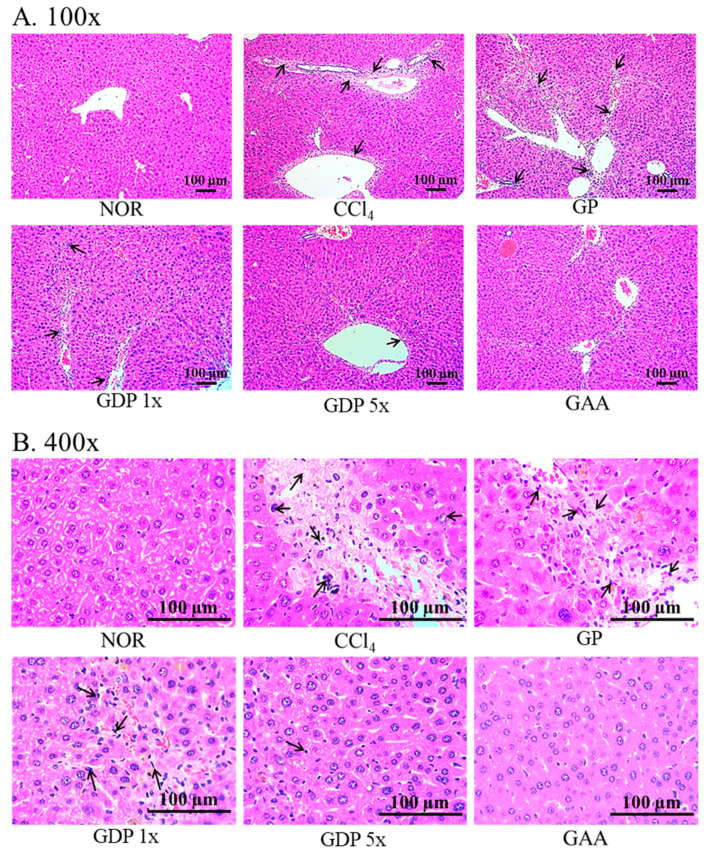
Histopathological evaluation of liver sections from CCl_4_-induced fibrotic mice following treatment with *Ganoderma amboinense* formulations. (**A**) Low-power (100×) and (**B**) high-power (400×) photomicrographs of hematoxylin and eosin-stained liver sections. Mice received intraperitoneal injections of olive oil (NOR) or CCl_4_ (0.4 µL/g) three times weekly for six weeks. Treatment groups (*n* = 7) were administered *G. amboinense* powder (GP; 410 mg/kg/day), sublingual dripping pills of *G. amboinense* fruiting body extract at 92.25 mg/kg/day (GDP 1×) or 461.25 mg/kg/day (GDP 5×), or purified ganoderic acid A (GAA; 1.07 mg/kg/day). Black arrows highlight hepatocellular necrosis, inflammatory cell infiltration, and disruption of hepatic cords. Scale bars = 100 µm.

**Figure 3:**
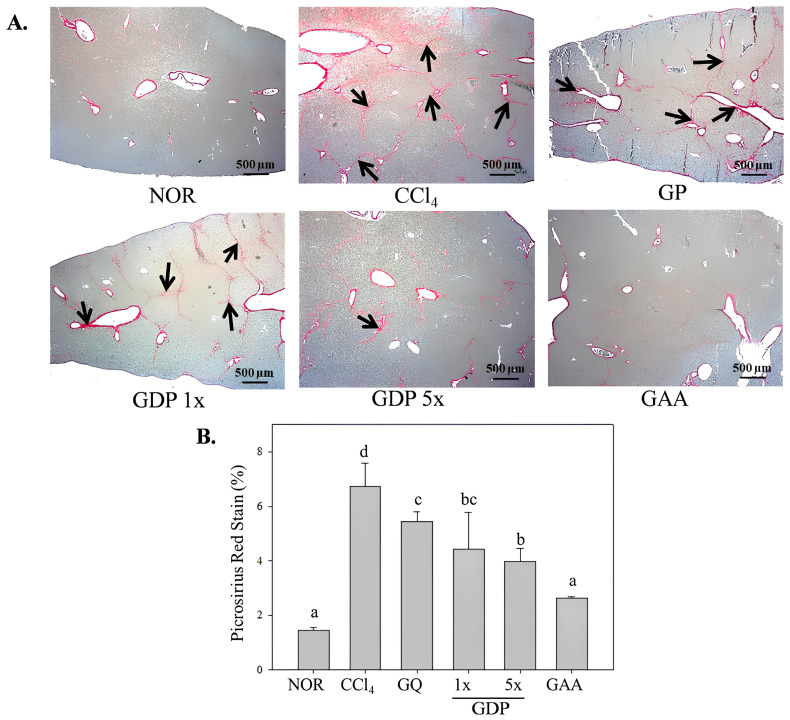
Picrosirius red staining of collagen deposition in liver sections from CCl_4_-induced fibrotic mice treated with *Ganoderma amboinense* formulations. (**A**) Representative liver micrographs (40× magnification; scale bar = 500 µm) of paraffin-embedded sections stained with picrosirius red. (**B**) Quantification of the picrosirius red–positive area as a percentage of total tissue section. Mice received intraperitoneal injections of olive oil (NOR) or CCl_4_ (0.4 µL/g) three times weekly for six weeks. Treatment groups (*n* = 7) were administered *G. amboinense* powder (GP; 410 mg/kg/day), sublingual dripping pills of *G. amboinense* fruiting body extract at 92.25 mg/kg/day (GDP 1×) or 461.25 mg/kg/day (GDP 5×), or purified ganoderic acid A (GAA; 1.07 mg/kg/day). Data are presented as mean ± SD (*n* = 7). Different letters indicate significantly different values according to a one-way ANOVA using Duncan’s multiple test (*p* < 0.05). Black arrows indicate collagen fiber deposition.

Hematoxylin and eosin staining (Figure 2) revealed that CCl_4_-induced disorganized hepatocyte architecture, cell membrane disruption, and nuclear shrinkage with focal necrosis. Treatment with GDP 5× and GAA substantially ameliorated these histopathological changes, restoring near-normal cellular morphology; GDP 1× provided partial protection, whereas GP showed minimal improvement. Picrosirius red staining (Figure 3) demonstrated extensive collagen deposition in the CCl_4_ group (*p* < 0.05 vs. NOR). Both GDP 5× and GAA treatments significantly reduced fibrotic area by approximately 60% (*p* < 0.05 vs. CCl_4_), with no significant difference between them; GDP 1× also reduced fibrosis significantly relative to GP (*p* < 0.05).

### 3.5. Hepatic Pro-Inflammatory Cytokine Expression

**Figure 4:**
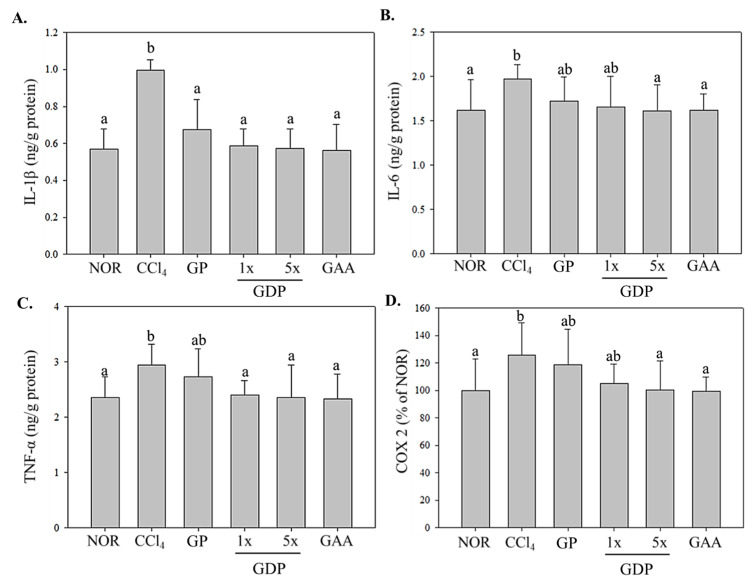
Hepatic pro-inflammatory cytokine and COX-2 expression in CCl_4_-induced fibrotic mice following treatment with *Ganoderma amboinense* formulations. After sacrifice, liver homogenates were assayed by ELISA for (**A**) interleukin-1β (IL-1β), (**B**) interleukin-6 (IL-6), (**C**) tumor necrosis factor-α (TNF-α), and (**D**) cyclooxygenase-2 (COX-2, expressed as % of NOR). Mice received intraperitoneal injections of olive oil (NOR) or CCl_4_ (0.4 µL/g) three times weekly for six weeks. Treatment groups (*n* = 7) were administered *G. amboinense* powder (GP; 410 mg/kg/day), sublingual dripping pills of *G. amboinense* fruiting body extract at 92.25 mg/kg/day (GDP 1×) or 461.25 mg/kg/day (GDP 5×), or purified ganoderic acid A (GAA; 1.07 mg/kg/day). Data are presented as mean ± SD (*n* = 7). Different letters indicate significantly different values according to a one-way ANOVA using Duncan’s multiple test (*p* < 0.05).

**Figure 5:**
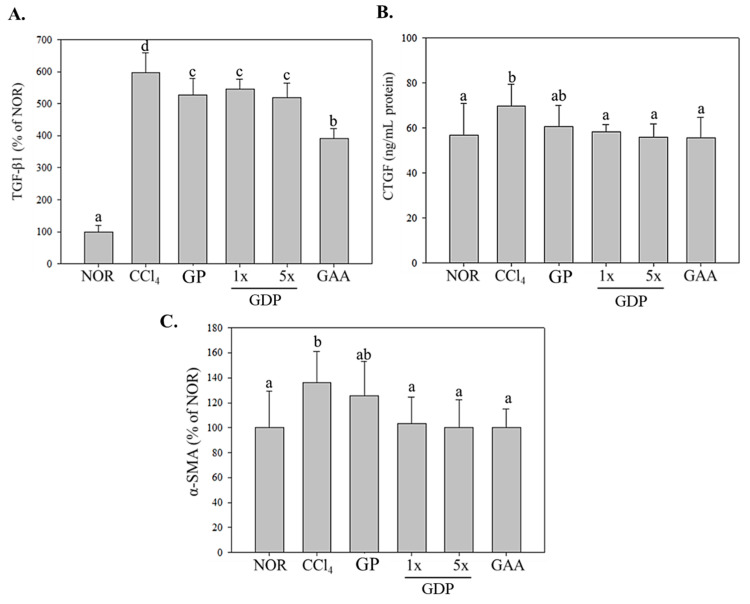
Inhibition of hepatic profibrotic marker expression in CCl_4_-induced fibrotic mice treated with *Ganoderma amboinense* formulations. (**A**) Transforming growth factor-β1 (TGF-β1) levels expressed as a percentage of the NOR control; (**B**) connective tissue growth factor (CTGF) concentration in ng/mL per g protein; (**C**) α-smooth muscle actin (α-SMA) expression expressed as a percentage of the NOR control. Mice received intraperitoneal injections of olive oil (NOR) or CCl_4_ (0.4 µL/g) three times weekly for six weeks. Treatment groups (*n* = 7) were administered *G. amboinense* powder (GP; 410 mg/kg/day), sublingual dripping pills of *G. amboinense* fruiting body extract at 92.25 mg/kg/day (GDP 1×) or 461.25 mg/kg/day (GDP 5×), or purified ganoderic acid A (GAA; 1.07 mg/kg/day). Data are presented as mean ± SD (*n* = 7). Different letters indicate significantly different values according to a one-way ANOVA using Duncan’s multiple test (*p* < 0.05).

CCl_4_ markedly upregulated hepatic IL-1β, IL-6, and TNF-α levels versus NOR (*p* < 0.05; Figure 4). GDP (1×, 5×) and GAA each normalized IL-1β to near-baseline levels (*p* < 0.05 vs. CCl_4_). IL-6 and TNF-α were significantly suppressed by GDP 5× and GAA (*p* < 0.05), while GDP 1× and GP displayed only non-significant trends. COX-2 expression was significantly reduced by GDP 5× and GAA (*p* < 0.05), with other groups showing downward trends (Figure 5).

### 3.6. Hepatic Pro-Fibrotic Marker Expression

CCl_4_-induced significant elevations in TGF-β1, CTGF, and α-SMA levels compared with the NOR group (*p* < 0.05; Figure 5), reflecting hepatic stellate cell activation and extracellular matrix accumulation. Both GDP doses (1× and 5×) and GAA significantly downregulated all three markers compared with CCl_4_ alone (*p* < 0.05), with GAA showing the greatest suppression of TGF-β1. In contrast, GP produced only non-significant reductions. These findings confirm that the novel dripping pill formulation delivers superior anti-fibrotic efficacy relative to unextracted powder and matches or exceeds the effects of purified ganoderic acid A, while the formulation excipients serve solely to protect active compounds from oxidative degradation rather than contribute additional pharmacological activity.

## 4. Discussion

Notably, Hsu et al. (2008) demonstrated that extracts of *G. amboinense* could protect mice from acetaminophen-induced acute liver injury, suggesting hepatoprotective potential [18]. Additional studies revealed that specific triterpenes isolated from *G. amboinense*, such as ganoderiol F and ganoderic acid X, exhibit antiproliferative effects and promote apoptosis in cancer cell lines [16,17]. These compounds act through mechanisms involving topoisomerase inhibition and cellular senescence, thereby indicating that *G. amboinense* harbors functionally potent metabolites distinct from those of *G. lucidum*. While both species produce lanostane triterpenoids, *G. lucidum* compounds have been more thoroughly investigated and commercialized. In contrast, *G. amboinense* possesses unique chemical scaffolds and therapeutic properties that are just beginning to be elucidated. Given the known influence of formulation on bioactivity, further investigation into optimal extraction conditions and delivery formats for *G. amboinense* is warranted. In this context, our one-pot ethanol extraction was intentionally optimized to co-recover ganoderic acid A (GA-A) and β-1,3-glucan, thereby enabling simultaneous enrichment of a potent antifibrotic triterpenoid and an immunomodulatory polysaccharide known to enhance antioxidant defense and tissue repair [30,31]. This dual-component recovery not only streamlines processing but also preserves potential synergistic effects between triterpenoid and polysaccharide fractions in liver protection [32]. This study contributes to bridging that knowledge gap by applying systematic extraction and characterization methodologies, highlighting the untapped pharmacological potential of this culturally significant yet scientifically overlooked species.

Dripping pills are an advanced dosage form widely applied in traditional Chinese medicine modernization due to their advantages in enhancing bioavailability, precise dosing, and patient compliance. Particularly in cardiovascular therapeutics, formulations such as Compound Danshen Dripping Pills (CDDP) have been shown to improve outcomes in patients with stable angina and myocardial ischemia by delivering multi-component agents with consistent pharmacokinetics and enhanced systemic exposure [21,22]. These benefits suggest that the dripping pill system could serve as a valuable delivery vehicle for other traditional medicinal materials, especially those rich in lipophilic compounds like triterpenoids.

*Ganoderma amboinense*, a rare and visually distinctive species of *Ganoderma* highly appreciated in East Asian cultures, contains a range of lanostane-type triterpenoids with potential bioactivity [11]. Despite its phytochemical richness, its pharmacological functions and delivery technologies remain underexplored. Unlike *Ganoderma lucidum*, which has been studied in powders, capsules, and aqueous extracts, there is no current research on the encapsulation of *Ganoderma* extracts—particularly from *G. amboinense*—into dripping pills for functional evaluation. To the best of our knowledge, this is the first study to formulate *Ganoderma* extract into a dripping pill dosage form and systematically evaluate its functional effects in vivo. By applying ethanol extraction and scale-up encapsulation, we were able to deliver a stable form of *G. amboinense* that demonstrates hepatoprotective efficacy in a CCl_4_-induced liver fibrosis mouse model. Our findings provide new evidence that the combination of traditional fungal medicine and modern drug delivery systems could yield promising functional health products, potentially expanding the clinical applications of medicinal mushrooms.

The present study demonstrates that the ethanol-extracted dripping pill formulation of *G. amboinense* fruiting body (GDP) confers superior protective effects against CCl_4_-induced hepatic fibrosis in mice compared to the unextracted powder form GP. Across all measured endpoints—body weight preservation, liver function markers (AST, ALT), histopathological structure, inflammatory cytokine expression, and pro-fibrotic marker suppression—the GDP groups (particularly the 5× dose) exhibited significantly greater efficacy than GP, which showed only marginal or non-significant trends. Notably, GDP mitigated CCl_4_-induced weight loss and hepatomegaly, significantly reducing liver weight and the liver-to-body weight ratio, while GP did not yield significant improvement. The hepatoprotective superiority of GDP was further evidenced by the normalization of serum AST and ALT activities, where the 5× formulation reduced both markers to levels comparable to those achieved by purified ganoderic acid A. In contrast, GP failed to significantly alter these enzymes, highlighting the limited bioactivity of the crude powder form.

The GDP formulation represents a pharmacologically optimized delivery system for *Ganoderma amboinense*, providing enhanced antifibrotic and anti-inflammatory effects over the crude powder and matching the efficacy of isolated ganoderic acid A. These results support the broader utility of dripping pills as a novel dosage form for lipophilic fungal constituents and validate the translational potential of *G. amboinense* in liver protection strategies. A key innovation in this formulation is the inclusion of red quinoa (*Chenopodium formosanum*) extract, rich in the bioflavonoid rutin, as an excipient during the dripping pill manufacturing process. Rutin has been demonstrated to exert anti-inflammatory effects via NF-κB and MAPK pathway inhibition in macrophage models (e.g., DH82 cells), and other studies have shown its hepatoprotective and anti-fibrotic roles through suppression of TLR4/P2X7/NF-κB signaling in hepatic models [33]. Additionally, rutin has been reported to alleviate liver injury by restoring redox balance and mitochondrial function in hepatocytes [34], further supporting its role as a functional stabilizer and synergistic component.

In this study, we demonstrated that *Ganoderma amboinense* extract formulated into a dripping pill dosage form (GDP) significantly attenuates CCl_4_-induced hepatic injury and fibrosis in mice. While previous studies have reported the hepatoprotective effects of ganoderic acids, especially ganoderic acid A (GAA), our results further establish the superior therapeutic potential of GDP compared to the raw powder form GP, both in serum biomarkers and histopathological features. Notably, the GDP 5× group outperformed the unprocessed powder group in preserving body weight, reducing liver-to-body weight ratio, and normalizing serum AST and ALT activities, indicating better systemic and hepatic protection. Histological analysis also revealed significant improvements in liver architecture and a marked reduction in collagen deposition in the GDP groups, comparable to purified GAA.

Histological examination supported these biochemical findings: GDP-treated livers maintained clearer hepatocyte architecture and exhibited markedly reduced necrosis and collagen deposition, with fibrotic areas diminished by ~60%. These improvements were absent or minimal in the GP group. At the molecular level, the dripping pill significantly suppressed hepatic expression of inflammatory mediators (IL-1β, IL-6, TNF-α, and COX-2) and fibrogenic markers (TGF-β1, CTGF, and α-SMA), while GP displayed only minor, non-significant reductions. The enhanced efficacy of the GDP formulation likely results from multiple factors: (1) improved solubility and absorption of bioactive lanostane triterpenoids due to ethanol extraction; and (2) sustained and controlled release enabled by the dripping pill matrix. Collectively, these features maximize the systemic bioavailability and pharmacological performance of *Ganoderma* compounds. In contrast, the unextracted powder lacks both bioactive enrichment and delivery optimization, resulting in subtherapeutic exposure. Moreover, GDP significantly suppressed the expression of hepatic pro-inflammatory cytokines, including IL-1β, TNF-α, IL-6, and COX-2, highlighting its anti-inflammatory effects. These outcomes are consistent with previous studies showing that triterpenoids from *Ganoderma* species downregulate NF-κB signaling and inflammatory mediators, contributing to reduced hepatic inflammation [35]. In particular, GDP 5× provided significant reductions in pro-fibrotic markers such as TGF-β1, CTGF, and α-SMA, which are critical mediators in hepatic stellate cell activation and extracellular matrix accumulation, underscoring its potential anti-fibrotic efficacy. To further contextualize these findings, the inhibition of TGF-β1 signaling observed with GDP parallels the mechanism targeted by clinical antifibrotic agents such as pirfenidone, which is currently approved for idiopathic pulmonary fibrosis and under investigation for hepatic fibrosis. While pirfenidone primarily attenuates TGF-β1–mediated fibroblast activation and collagen synthesis, our results show that GDP (particularly at the 5× dose) significantly reduced hepatic TGF-β1 expression together with downstream fibrogenic markers (CTGF, α-SMA). This suggests that GDP exerts comparable pathway-level modulation. Although direct head-to-head efficacy studies are required, the present findings highlight GDP as a natural multi-component alternative that may synergize with, or provide complementary benefits to, existing antifibrotic pharmacotherapies.

To our knowledge, this is the first study to evaluate the functional efficacy of *Ganoderma amboinense* ethanol extract delivered via a dripping pill system, an innovative dosage form that enhances active compound stability, improves bioavailability, and ensures consistent dosing. While dripping pills have been extensively used in traditional Chinese medicine for cardiovascular and metabolic disorders [36], their application in fungal medicine remains unexplored until now. The current findings suggest that GDP, supported by the bioactivity of red quinoa-derived rutin, represents a promising delivery platform for fungal triterpenoids in hepatic health applications. Future studies should directly evaluate the pharmacokinetics and safety profile of the GDP formulation in preclinical settings to substantiate its potential for further development into functional or therapeutic products.

## 5. Conclusions

This study is the first to demonstrate that an ethanol-extracted, sublingual dripping pill formulation of *Ganoderma amboinense* fruiting body (GDP) effectively attenuates CCl_4_-induced liver injury and fibrosis in vivo. At both the human equivalent (1×) and five-fold (5×) doses, GDP significantly preserved body weight and liver index, normalized serum AST and ALT activities, and reversed histopathological damage. Moreover, GDP robustly suppressed hepatic expression of key pro-inflammatory cytokines (IL-1β, IL-6, TNF-α, COX-2) and pro-fibrotic markers (TGF-β1, CTGF, α-SMA), matching or exceeding the efficacy of purified ganoderic acid A and markedly outperforming unprocessed *G. amboinense* powder. These results confirm that the dripping pill dosage form provides enhanced delivery and bioactivity of fungal triterpenoids, offering a novel, practical platform for developing liver-protective nutraceuticals and functional foods from *G. amboinense*.

## Figures and Tables

**Table 1:** Effects of normal control, fibrosis control, and different *Ganoderma amboinense* preparations on body weight, liver weight, and liver weight-to-body weight ratio in CCl_4_-induced liver fibrosis mice.

Groups	Body Weight (g)	Liver Weight (g)	Liver Weight/Body Weight (%)
NOR	27.6 ± 0.8 ^d^	1.2 ± 0.05 ^a^	4.71 ± 0.27 ^a^
CCl_4_	23.7 ± 1.7 ^a^	1.7 ± 0.02 ^c^	6.47 ± 0.42 ^c^
GP	24.9 ± 2.0 ^ab^	1.3 ± 0.13 ^ab^	5.47 ± 0.18 ^b^
GDP 1x	25.9 ± 1.1 ^bc^	1.3 ± 0.18 ^ab^	5.70 ± 0.21 ^b^
GDP 5x	26.1 ± 0.7 ^bcd^	1.4 ± 0.14 ^ab^	5.44 ± 0.33 ^b^
GAA	26.9 ± 1.1 ^cd^	1.4 ± 0.12 ^b^	5.68 ± 0.25 ^b^

Mice received intraperitoneal injections of olive oil (NOR) or CCl_4_ (0.4 µL/g) three times weekly for six weeks. Treatment groups (*n* = 7) were administered *G. amboinense* powder (GP; 410 mg/kg/day), sublingual dripping pills of *G. amboinense* fruiting body extract at 92.25 mg/kg/day (GDP 1×) or 461.25 mg/kg/day (GDP 5×), or purified ganoderic acid A (GAA; 1.07 mg/kg/day). Data are presented as mean ± SD (*n* = 7). Different letters indicate significantly different values according to a one-way ANOVA using Duncan’s multiple test (*p* < 0.05); values sharing the same letter are not significantly different.

**Table 2:** The effects of extracted dripping pills of *Ganoderma amboinense* on AST and ALT in serum of mice with CCl_4_-induced liver fibrosis.

Groups	AST Activity (U/L)	ALT Activity (U/L)
NOR	71.9 ± 5.0 ^a^	37.7 ± 1.8 ^a^
CCl_4_	84.1 ± 8.8 ^b^	59.1 ± 3.8 ^d^
GP	78.1 ± 8.8 ^ab^	54.7 ± 5.9 ^cd^
GDP 1 x	74.7 ± 5.6 ^a^	52.7 ± 3.9 ^c^
GDP 5 x	71.9 ± 4.1 ^a^	46.6 ± 6.6 ^b^
GAA	76.1 ± 4.5 ^a^	51.1 ± 4.9 ^bc^

Mice received intraperitoneal injections of olive oil (NOR) or CCl_4_ (0.4 µL/g) three times weekly for six weeks. Treatment groups (*n* = 7) were administered *G. amboinense* powder (GP; 410 mg/kg/day), sublingual dripping pills of *G. amboinense* fruiting body extract at 92.25 mg/kg/day (GDP 1×) or 461.25 mg/kg/day (GDP 5×), or purified ganoderic acid A (GAA; 1.07 mg/kg/day). Data are presented as mean ± SD (*n* = 7). Different letters indicate significantly different values according to a one-way ANOVA using Duncan’s multiple test (*p* < 0.05).

## Data Availability

All data included in this study are available upon request by contacting the corresponding authors.
